# Central command increases muscular oxygenation of the non‐exercising arm at the early period of voluntary one‐armed cranking

**DOI:** 10.14814/phy2.13237

**Published:** 2017-04-05

**Authors:** Kei Ishii, Kanji Matsukawa, Ryota Asahara, Nan Liang, Kana Endo, Mitsuhiro Idesako, Kensuke Michioka, Yu Sasaki, Hironobu Hamada, Kaori Yamashita, Tae Watanabe, Tsuyoshi Kataoka, Makoto Takahashi

**Affiliations:** ^1^ Department of Integrative PhysiologyGraduate School of Biomedical and Health SciencesHiroshima UniversityHiroshimaJapan; ^2^ Automotive Human Factors Research CenterNational Institute of Advanced Industrial Science and TechnologyTsukubaJapan; ^3^ Department of Physical Analysis and Therapeutic SciencesGraduate School of Biomedical and Health SciencesHiroshima UniversityHiroshimaJapan; ^4^ Department of Health Care for AdultsGraduate School of Biomedical and Health SciencesHiroshima UniversityHiroshimaJapan; ^5^ Department of BiomechanicsGraduate School of Biomedical and Health SciencesHiroshima UniversityHiroshimaJapan

**Keywords:** Central command, cholinergic and *β*‐adrenergic vasodilatation, exercise, near‐infrared spectroscopy, proximal and distal arm muscles

## Abstract

This study aimed to examine whether central command increases oxygenation in non‐contracting arm muscles during contralateral one‐armed cranking and whether the oxygenation response caused by central command differs among skeletal muscles of the non‐exercising upper limb. In 13 male subjects, the relative changes in oxygenated‐hemoglobin concentration (Oxy‐Hb) of the non‐contracting arm muscles [the anterior deltoid, triceps brachii, biceps brachii, and extensor carpi radialis (ECR)] were measured during voluntary one‐armed cranking (intensity, 35–40% of maximal voluntary effort) and mental imagery of the one‐armed exercise for 1 min. Voluntary one‐armed cranking increased (*P *<* *0.05) the Oxy‐Hb of the triceps, biceps, and ECR muscles to the same extent (15 ± 4% of the baseline level, 17 ± 5%, and 16 ± 4%, respectively). The greatest increase in the Oxy‐Hb was observed in the deltoid muscle. Intravenous injection of atropine (10–15 *μ*g/kg) and/or propranolol (0.1 mg/kg) revealed that the increased Oxy‐Hb of the arm muscles consisted of the rapid atropine‐sensitive and delayed propranolol‐sensitive components. Mental imagery of the exercise increased the Oxy‐Hb of the arm muscles. Motor‐driven passive one‐armed cranking had little influence on the Oxy‐Hb of the arm muscles. It is likely that central command plays a role in the initial increase in oxygenation in the non‐contracting arm muscles via sympathetic cholinergic vasodilatation at the early period of one‐armed cranking. The centrally induced increase in oxygenation may not be different among the distal arm muscles but may augment in the deltoid muscle.

## Introduction

The presence of a neurally mediated vasodilator mechanism for blood vessels in skeletal muscle has been suggested not only in animals but in humans (Blair et al. 1959; Abrahams et al. 1960; Shepherd 1983; Dietz et al. 1994; Matsukawa et al. 2013). In some animal species, it is considered that efferent signals from the specific brain areas (such as the lateral hypothalamus and midbrain periaqueductal gray matter and ventral tegmentum area) cause muscle vasodilatation via activation of sympathetic cholinergic fibers that are normally silent (Schramm and Bignall 1971; Horeyseck et al. 1976; Dean and Coote 1986; Matsukawa et al. 1997, 2011). In humans, it is known that vasodilatation in the non‐exercising forearm occurs at the early period of isometric handgrip or finger exercise of the contralateral limb, whereas such exercise does not increase blood flow to the non‐exercising leg (Eklund et al. 1974; Rusch et al. 1981; Duprez et al. 1989). The initial vasodilatation in non‐exercising forearm was atropine‐sensitive (Sanders et al. 1989), suggesting existence of the sympathetic cholinergic vasodilator mechanism in humans. However, it remains unknown whether the neurally mediated vasodilatation occurs similarly among skeletal muscles of the non‐exercising upper limb. In addition, since Lusina et al. (2008) reported that a decrease in oxygenation of the contracting deltoid muscle during progressive arm cranking was smaller than the decrease in the contracting triceps and biceps muscles, the increased oxygenation response may differ among the individual arm muscles.

The mechanism(s) responsible for the neurally mediated vasodilatation of the non‐exercising upper limb has not been determined yet. A series of our human studies (Ishii et al. 2012, 2013, 2014, 2016) demonstrated the initial cholinergic vasodilation and increased oxygenation in the non‐contracting vastus lateralis muscle during voluntary one‐legged cycling but not passive cycling. The evidence suggests that the initial vasodilator and oxygenation responses are evoked by a descending signal from higher brain centers (termed central command), rather than by activation of mechanosensitive afferents in contracting muscles (termed muscle mechanoreflex). Along this line, we hypothesized that oxygen supply to non‐contracting arm muscles may increase during contralateral dynamic exercise, via a centrally induced cholinergic vasodilator mechanism, and that the centrally induced increased oxygenation may differ among skeletal muscles of the non‐exercising upper limb. To test the hypotheses, using near‐infrared spectroscopy (NIRS), the relative concentrations of oxygenated‐ and deoxygenated‐hemoglobin (Oxy‐ and Deoxy‐Hb) in non‐contracting arm muscles were measured during voluntary and passive one‐armed cranking and during mental imagery of the voluntary arm exercise. Both central command and muscle mechanoreflex are activated by voluntary exercise, while passive limb movement activates selectively muscle mechanoreflex (McDaniel et al. 2010; Ishii et al. 2012). Motor imagery is supposed to simulate central control of the cardiovascular system during exercise without any afferent feedback from contracting muscles (Decety et al. 1993; Williamson et al. 2002; Ishii et al. 2012). Furthermore, we examined the influences of cholinergic and *β*‐adrenergic vasodilatation on the Oxy‐Hb responses of the arm muscles during voluntary one‐armed cranking using muscarinic and *β*‐adrenergic blockades.

## Methods

### Subjects

Thirteen healthy male subjects (age, 24 ± 1 years; height, 175 ± 2 cm; body weight, 68 ± 3 kg) participated in this study. None of the subjects suffered from any known cardiovascular and neuromuscular disease. They did not take any medications. The experimental procedures and protocols were performed in accordance with the *Declaration of Helsinki* and approved by the Institutional Ethical Committee. The subjects gave their informed written consent prior to the experiments. All experiments were performed in a soundproof room, in which temperature was maintained 24–26°C.

### One‐armed cranking exercise

**Figure 1:**
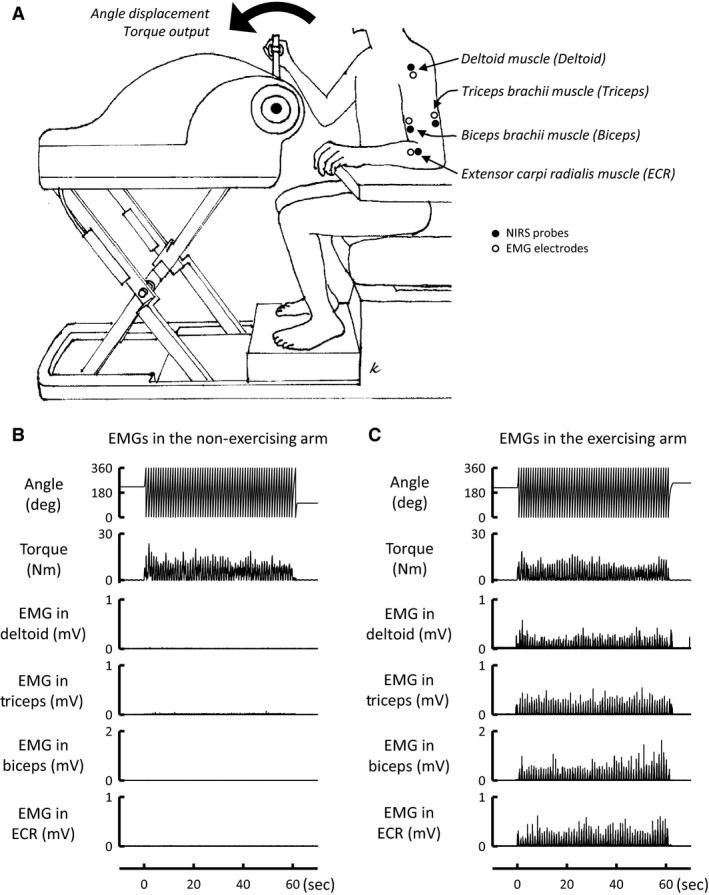
(A) Experimental setup. (B) Representative recordings of the crank angular displacement and developed torque of the ergometer and integrated electromyogram (EMG) signals of the non‐contracting arm muscles during 1 min of voluntary one‐armed cranking in a subject. (C) Representative recordings of the integrated EMG signals of the contracting arm muscles during the exercise in the same subject. The EMG signals of the non‐contracting and contracting muscles were not simultaneously recorded. EMG activity was recorded in the anterior deltoid (Deltoid), triceps brachii (Triceps), biceps brachii (Biceps), and extensor carpi radialis (ECR) muscle.

One‐armed cranking exercise with the right arm was performed for 1 min at 60 rpm in an upright sitting posture on a seat of a specially designed cycle ergometer (Strength Ergo 240 BK‐ERG‐003, Mitsubishi Electric Engineering, Tokyo, Japan) as shown in Figure 1. The positions of the crank and seat were adjusted so that the subjects remained in a comfortable and certain posture. Torque against the wheel shaft and angular displacement of the ergometer crank were continuously measured. The subjects performed two types of one‐armed cranking: voluntary and passive mode. In a voluntary trial, the subjects were given a verbal instruction of “please start exercise whenever you want after you calm down and rest sufficiently,” and then they started exercise arbitrarily without any cue. In a passive trial, one‐armed cranking movement was driven by a motor of the ergometer without any verbal cue and volitional effort. To minimize a chance to anticipate the start of passive movement, when the passive movement would start was not informed to the subjects. On a separate day prior to the main experiment, the subjects were familiarized to one‐armed cranking in the laboratory environment and performed an incremental one‐armed exercise test to determine the maximal voluntary effort (MVE) as previously reported (Ishii et al. 2012). The rating of perceived exertion (RPE) was asked after each bout of exercise, according to the Borg 6‐20 unit scale (Borg 1970).

### Mental imagery of one‐armed cranking

Mental imagery of voluntary one‐armed cranking (cranking imagery) for 1 min was performed to examine the central influence on muscle oxygenation without any feedback from contracting muscle. Immediately after a verbal cue was given, the subjects were instructed to imagine voluntary one‐armed cranking that they performed. As control, they were instructed to imagine a circle shape with no relation to exercise (circle imagery). The vividness score of imagery [from 0 (not vivid at all) to 10 (the most vivid)] was asked after each imagery task as previously reported (Williamson et al. 2002; Ishii et al. 2012).

### Measurements of muscle oxygenation

NIRS was utilized for measuring the Oxy‐ and Deoxy‐Hb of non‐contracting muscles. The basic principle of NIRS is that near‐infrared light from three laser photodiodes with different wavelengths penetrates skeletal muscle tissue and some of the light is absorbed by Hb, myoglobin (Mb), and cytochromes and that the remaining light scattered by the tissue is picked up with photo‐detectors (Boushel and Piantadosi 2000; McCully and Hamaoka 2000). It has been indicated that the signals of NIRS are chiefly affected by the Hb in blood vessels of muscle tissue rather than Mb and cytochromes (Seiyama et al. 1988). NIRS does not directly measure blood flow but provides information for a balance of oxygen supply and utilization in the microcirculation within the illuminated tissue (Mancini et al. 1994; Barrett and Rattigan 2012). As long as oxygen utilization of a muscle remains constant, changes in Oxy‐Hb will reflect changes in oxygen supply. Indeed, the responses in Oxy‐Hb and limb blood flow correlated each other during reflexly and pharmacologically evoked vasoconstriction (Fadel et al. 2004; Ives et al. 2014) and during contralateral leg exercise (Mizuno et al. 2006). Hoshi et al. (2001) demonstrated that, in a perfused rat brain model, increasing blood flow always caused a rise in Oxy‐Hb and a reduction in Deoxy‐Hb, while decreasing blood flow was accompanied by a decrease in Oxy‐Hb with various changes in Deoxy‐Hb.

A pair of photo‐emission and photo‐detection probes was placed on various muscles of the left arm [anterior deltoid, triceps brachii, biceps brachii, and extensor carpi radialis (ECR)] so that near‐infrared light would intersect with muscle bundles (Fig. 1). The interprobe distance was 4 cm for the deltoid and triceps muscles and 3 cm for the biceps and ECR muscles, respectively. The near‐infrared light scattered through muscle tissue was sampled at a rate of 6 Hz (NIRO 200, Hamamatsu Photonics, Hamamatsu, Japan) or 10 Hz (PocketNIRS Duo, DynaSense Inc., Hamamatsu, Japan) and converted to optical densities with the near‐infrared spectrometers. The absolute concentrations of Oxy‐ and Deoxy‐Hb could not be obtained, because the pathlength of near‐infrared light within tissue was unknown in vivo. So the relative changes in the NIRS signals against the pretask values were obtained in each trial. In addition, the relative percent changes in Oxy‐Hb of the triceps, biceps, and ECR muscles were calculated against the baseline control that was defined as a difference between the Oxy‐Hb values during the pretask period and during suprasystolic inflation of a pneumatic cuff wrapped around the upper arm (Tables 1 and 2). In some subjects, the baseline Oxy‐Hb control could not be obtained because of the two reasons: (1) they moved accidentally the left arm (where NIRS probes were set) and/or (2) baseline NIRS data drifted gradually throughout the experiments, possibly due to an influence of hydrostatic pressure.

**Table 1:** Effects of atropine and subsequent propranolol on the baseline values and the rating of perceived exertion (RPE) for voluntary one‐armed cranking

	Control	Atropine	Atropine and propranolol
HR (beats/min)	76 ± 3	110 ± 4^a^	85 ± 1^a^^,^ b
SV (mL)	63 ± 4	48 ± 2^a^	47 ± 2^a^
CO (L/min)	4.7 ± 0.2	5.2 ± 0.2^a^	4.0 ± 0.2^a^^,^ b
MAP (mmHg)	98 ± 4	100 ± 3	96 ± 3
TPR (mmHg/L/min)	21.1 ± 1.2	19.5 ± 0.6	24.9 ± 1.8^a^^,^ b
Oxy‐Hb in Triceps (*μ*Mcm)	539 ± 89 (*n* = 7)	550 ± 87 (*n* = 7)	535 ± 101 (*n* = 6)
Oxy‐Hb in Triceps (%)	100	102 ± 1	94 ± 3^b^
Oxy‐Hb in Biceps (*μ*Mcm)	443 ± 56 (*n* = 6)	451 ± 57 (*n* = 6)	442 ± 64 (*n* = 5)
Oxy‐Hb in Biceps (%)	100	102 ± 1	96 ± 2^b^
Oxy‐Hb in ECR (*μ*Mcm)	387 ± 45 (*n* = 6)	403 ± 50 (*n* = 6)	375 ± 68 (*n* = 4)
Oxy‐Hb in ECR (%)	100	104 ± 2	101 ± 2
RPE (Borg score)	12.6 (12–13)	12.9 (12–13)	13.5 (13–14)

Values are means ± SE. RPE are means (interquartile range).

HR, heart rate; SV, stroke volume; CO, cardiac output; MAP, mean arterial blood pressure; TPR, total peripheral resistance; Oxy‐Hb, oxygenated‐hemoglobin; Baseline raw and %values of Oxy‐Hb were calculated using the minimum value obtained by a cuff inflation. The baseline cardiovascular variables were obtained in 10 subjects, while the baseline Oxy‐Hb values were obtained in 4–7 subjects.

a Significant difference (*P *<* *0.05) from the control condition.

b Significant difference (*P *<* *0.05) between the conditions (atropine vs. atropine and propranolol).

**Table 2:** Effects of propranolol and subsequent atropine on the baseline values and the RPE for voluntary one‐armed cranking

	Control	Propranolol	Propranolol and atropine
HR (beats/min)	75 ± 4	63 ± 2^a^	86 ± 3^a^^,^ b
SV (mL)	71 ± 6	70 ± 4	55 ± 3^a^^,^ b
CO (L/min)	5.3 ± 0.4	4.3 ± 0.2^a^	4.6 ± 0.2^a^
MAP (mmHg)	96 ± 4	93 ± 3	93 ± 3
TPR (mmHg/L/min)	18.8 ± 1.2	22.1 ± 1.1^a^	20.6 ± 1.1^a^^,^ b
Oxy‐Hb in Triceps (*μ*Mcm)	498 ± 98 (*n* = 7)	441 ± 88 (*n* = 7)	449 ± 86 (*n* = 7)
Oxy‐Hb in Triceps (%)	100	89 ± 2^a^	91 ± 2^a^
Oxy‐Hb in Biceps (*μ*Mcm)	460 ± 37 (*n* = 9)	436 ± 36 (*n* = 9)	459 ± 31 (*n* = 9)
Oxy‐Hb in Biceps (%)	100	95 ± 2	101 ± 3
Oxy‐Hb in ECR (*μ*Mcm)	381 ± 39 (*n* = 8)	349 ± 39 (*n* = 8)	384 ± 37 (*n* = 8)
Oxy‐Hb in ECR (%)	100	91 ± 2^a^	102 ± 3^b^
RPE (Borg score)	12.8 (12–13)	13.5 (13–14)	13.9 (13–15)^b^

Values are means ± SE. RPE are means (interquartile range).

The baseline raw and %values of Oxy‐Hb were calculated using the minimum value obtained by a cuff inflation. The baseline cardiovascular variables were obtained in 10 subjects, while the baseline Oxy‐Hb values were obtained in 7–9 subjects. RPE, rating of perceived exertion; TPR, total peripheral resistance.

a Significant difference (*P *<* *0.05) from the control condition.

b Significant difference (*P *<* *0.05) between the conditions (propranolol vs. propranolol and atropine).

### Cardiovascular and electromyogram recordings

An electrocardiogram (ECG) was monitored with a telemetry system (DynaScope DS‐3140, Fukuda Denshi, Tokyo, Japan). Arterial blood pressure (AP) was noninvasively and continuously measured with a Finometer^®^ (Finapres Medical Systems BV, Arnhem, the Netherlands), whose cuff was attached to the left middle or index finger. The AP waveform was sampled at a frequency of 200 Hz. The beat‐to‐beat values of mean AP (MAP) and heart rate (HR) were obtained throughout the experiments. Simultaneously, the beat‐to‐beat values of cardiac output (CO), stroke volume (SV), and total peripheral resistance (TPR) were calculated from aortic pressure waveform by using a Modelflow^®^ software (BeatScope 1.1, Finapres Medical Systems BV, Arnhem, the Netherlands).

Electromyogram (EMG) activity of the non‐contracting arm muscles was measured using a pair of silver‐bar electrodes attached near the probes of NIRS (Bagnoli‐2 and 4 EMG Systems, Delsys, Boston, MA). EMG activity was also recorded in the contracting arm muscles in four subjects. The EMG signals were amplified (×10000) and passed through a bandpass filter between 20 and 2000 Hz.

### Experimental protocols

**Figure 2:**
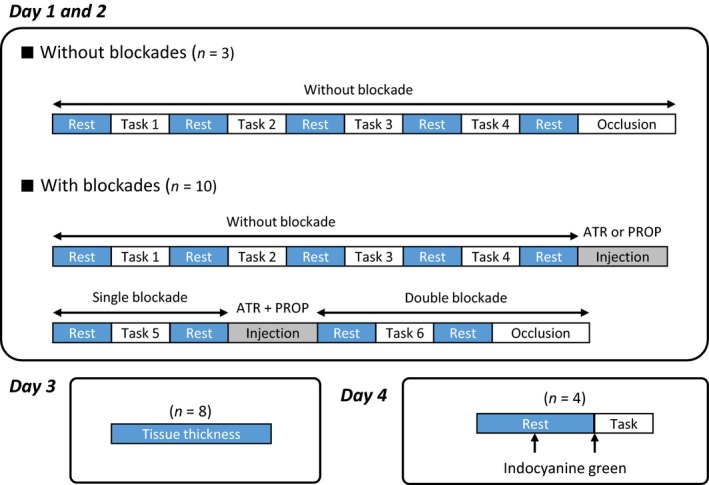
A time schedule of the experiments. On the day 1 and 2, ten of the 13 subjects performed individual tasks (voluntary one‐armed cranking with 35–40% of the maximal voluntary effort, passive one‐armed cranking, cranking imagery, and circle imagery) before and after autonomic blockades. Three of the 13 subjects performed the tasks without autonomic blockades. The order of the tasks was randomized, although the cranking imagery task always followed the voluntary cranking task. The tissue thickness under the NIRS probes was measured on the day 3 after the main experiment. A transit time of indocyanine green dye from the left cephalic vein to the left fingertip was measured during resting and at the early period of exercise on the day 4.

A time schedule of the experiments is shown in Figure 2. On the day 1 and 2, ten of the 13 subjects performed individual tasks before and after autonomic blockades; three subjects did not receive any autonomic blockade. The tissue thickness under the NIRS probes was measured on the day 3 after the main experiments. A transit time of indocyanine green dye from the left cephalic vein to the left fingertip was measured during resting and at the early period of exercise on the day 4. The details of each protocol are described as follows.

#### Days 1 and 2: muscle oxygenation measurements

In the absence of any drug (control condition), all 13 subjects performed (1) voluntary one‐armed cranking with 35–40% of the MVE, (2) passive one‐armed cranking, (3) cranking imagery, and (4) circle imagery. The order of the tasks was randomized, although a cranking imagery trial always followed a voluntary cranking trial. Subsequently, in 10 of the 13 subjects, atropine sulfate (10–15 *μ*g/kg) or propranolol hydrochloride (0.1 mg/kg) was intravenously administrated into the left cephalic vein. After a rest period from the first injection, voluntary one‐armed cranking was performed under cholinergic or *β*‐adrenergic blockade (atropine or propranolol condition). Subsequently, the first infused drug (at one‐third of the initial dose) and the other drug were conjointly injected. After a rest period from the subsequent second injection, voluntary cranking was performed again under both cholinergic and *β*‐adrenergic blockades (atropine and propranolol condition). The reverse order of the drug infusions was conducted on a separate day after more than 1 week from the first experiment day. We confirmed that there were no or negligible EMG activities (less than 5 *μ*V) of the non‐contracting arm muscles in all interventions (as represented in Fig. 1). At the end of the experiments, a pneumatic cuff wrapped around the upper arm was inflated with a pressure of 200–250 mmHg to obtain the minimum Oxy‐Hb values in the triceps, biceps, and ECR muscles.

Both atropine and propranolol have the same half‐life period of approximately 4 h (Brown and Taylor 2001; Hoffman 2001). We observed that intravenous administration of atropine or propranolol significantly affected the baseline values of the cardiovascular variables throughout the experiments and atropine caused pupil dilatation and thirsting. Although atropine and propranolol administered systemically may enter into the central nervous system, the dose of atropine administered in this study was as low as to have almost no detectable effect on the central nervous system (Brown and Taylor 2001) and the small dose of propranolol did not alter the resting and response value of MSNA during lower body negative pressure (Jacobsen et al. 1992) and the RPE during exercise in this study.

#### Day 3: tissue thickness measurements

On a separate day after the main experiment, tissue thickness under the NIRS probes was measured using an ultrasound Doppler instrument (Vivid S5, GE Healthcare, Tokyo, Japan) in eight subjects. The thickness of skin and fat tissues under the NIRS probes was not significantly different among the individual sites (3.3 ± 0.3 mm for the deltoid, 3.3 ± 0.3 mm for the triceps, 3.6 ± 0.4 mm for the biceps, and 2.7 ± 0.2 mm for the ECR). The thickness from the surface to the deepest edge of the target muscle tissue was 17.9 ± 0.8 mm for the deltoid, 18.3 ± 1.0 mm for the triceps, 20.1 ± 1.8 mm for the biceps, and 12.3 ± 0.9 mm for the ECR. Since the near‐infrared light travels in an arch shape and reaches into a depth of about one‐half of the interprobe distance (3 or 4 cm) (McCully and Hamaoka 2000), the near‐infrared light seemed to pass through the individual target muscle tissues sufficiently. Therefore, the NIRS signal was likely to reflect chiefly the changes in oxygenation of the target muscle tissue as well as skin and fat tissues above the muscle.

#### Day 4: transit time measurement

To estimate the transit time of epinephrine from the adrenal medulla to arterial vessels of the upper limb, we measured a dye dilution curve of the first pass of injected indocyanine green with a dye densitometry analyzer (DDG‐3300, Nihon Kohden, Tokyo, Japan) in four subjects on a separate day after the main experiments. A pulse dye‐densitometry probe was attached at the left fingertip. Indocyanine green (5 mg/mL) was injected into the left cephalic vein and flushed with a bolus of saline during resting and at the early period of voluntary one‐armed cranking. A transit time interval of the dye from the left cephalic vein to the left fingertip was calculated from the dye dilution curve. At rest, the mean time interval was ~37 sec (range, 26–55 sec), while the mean time interval during voluntary one‐armed cranking was ~32 sec (range, 24–38 sec).

### Data and statistical analyses

The data of the developed torque and crank displacement of the ergometer, AP, ECG, EMG, and NIRS signals were stored to computers at a sampling frequency of 1000 Hz (MP150, BIOPACK Systems, Santa Barbara, CA and PowerLab 16/35, ADInstruments‐Japan, Nagoya, Japan) for off‐line analysis. The onset of task was defined as “time = 0” according to the onset of crank displacement in the cranking task and the onset of a marker signal for the verbal cue in the imagery task. The baseline values were defined as the averages in the pretask resting period (for more than 10 sec) in each intervention. In voluntary exercise, the 10 sec‐period prior to the onset of exercise was excluded from the baseline. The baseline values were compared among the three conditions [control (without any drug) versus atropine or propranolol versus atropine and propranolol] by a one‐way ANOVA with repeated measures and a Holm‐Sidak post hoc test. The changes in cardiovascular and NIRS signals were sequentially averaged every 1 sec. The individual drug‐sensitive components of the Oxy‐Hb responses were determined by subtracting the Oxy‐Hb response with atropine or propranolol from the Oxy‐Hb response without any drug and by subtracting the Oxy‐Hb response with the combined drugs from the Oxy‐Hb response with atropine or propranolol, respectively. To assess the significant changes in each variable from the baseline control, a one‐way ANOVA with repeated measures was used with a Holm‐Sidak post hoc test. The time interval of the dye from the left cephalic vein to the left fingertip suggests that if epinephrine was secreted at the onset of exercise, the secreted epinephrine will reach forearm at approximately 32 sec (range, 24–38 sec) after the exercise onset. Based on this finding, the cardiovascular and NIRS responses during the early (10–20 sec; before epinephrine arrival) and later (40–60 sec; after epinephrine arrival) periods of cranking exercise were compared between the conditions (control vs. atropine or propranolol vs. atropine and propranolol) using a two‐way ANOVA with repeated measures and a Holm‐Sidak post hoc test. In the imagery tasks, the response of each variable was obtained as an average over a time period from 30 to 45 sec after the imagery onset as previously reported (Ishii et al. 2012, 2013, 2014). The Oxy‐Hb responses during actual exercise or motor imagery were compared among the individual muscles using a one‐way ANOVA with a Holm‐Sidak post hoc test. The comparison of the Borg scales and the vividness scores between the conditions was conducted using a Friedman test or a Wilcoxon rank sum test. A level of statistical significance was defined at *P *<* *0.05 in all cases. All statistical analyses were performed using SigmaPlot^®^ version 12.5 (Systat Software, San Jose, CA). The cardiovascular and NIRS variables are expressed as means ± SE. The Borg scales and vividness scores are expressed as means (interquartile range).

## Results

### The muscle oxygenation responses to voluntary versus passive arm exercise

The baseline values of the cardiovascular variables without any drug are summarized in Tables 1 and 2. HR, SV, and CO increased (*P *<* *0.05) at the early period of voluntary one‐armed cranking. The increases were sustained throughout the exercise (34 ± 2 beats/min, 16 ± 1 mL, and 3.6 ± 0.2 mL/min, respectively). MAP increased gradually 34 ± 4 mmHg until the end of the exercise. The decrease in TPR occurred initially and then the decrease became smaller during the later period of exercise. Mean RPE was 13 (interquartile range, 12–14).

**Figure 3:**
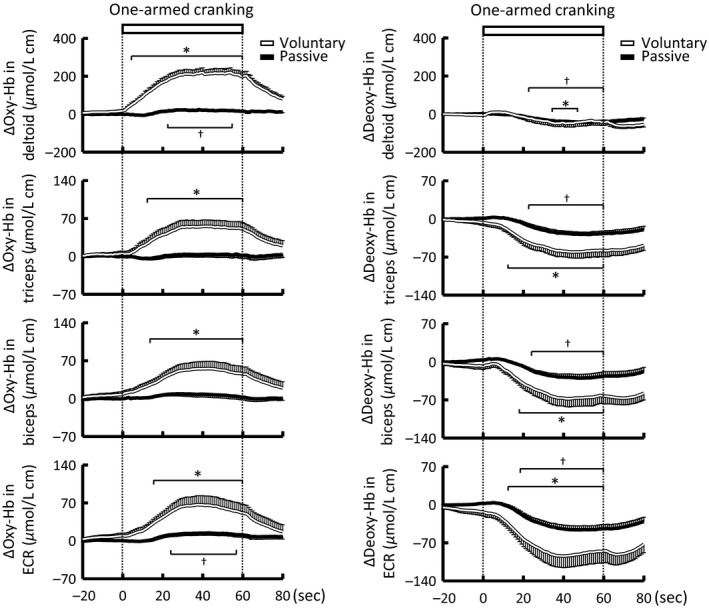
Time courses of the oxygneated‐ and deoxygenated‐hemoglobin (Oxy‐ and Deoxy‐Hb) responses in the non‐contracting arm muscles during voluntary (white lines) and passive one‐armed cranking (black lines) in 13 subjects. Each variable was sequentially calculated every 1 sec. Values are mean ± SE. * Significant difference (*P *<* *0.05) from the baseline before voluntary exercise. † Significant difference (*P *<* *0.05) from the baseline before passive exercise.

**Figure 4:**
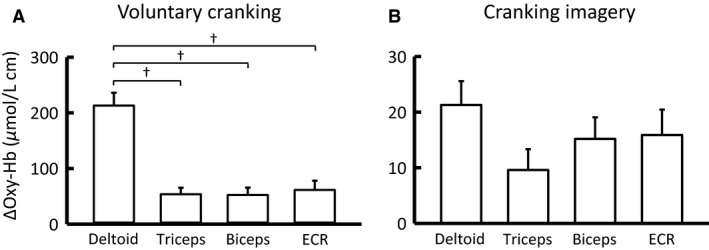
Comparison in the average Oxy‐Hb responses among the non‐contracting arm muscles during voluntary one‐armed cranking (A) and cranking imagery (B) in 13 subjects. Values are mean ± SE. † Significant difference (*P *<* *0.05) between the muscles. In both voluntary cranking exercise and cranking imagery, any of the Oxy‐Hb responses was significant from the baseline level.

Figures 3 and 4 summarize the time courses and magnitudes of the Oxy‐Hb responses in the non‐contracting arm muscles during voluntary one‐armed cranking. The Oxy‐Hb of the four arm muscles increased (*P *<* *0.05) at the early period of exercise and reached the peak approximately at the middle of the exercise period. The Oxy‐Hb response in the deltoid muscle was much greater (*P *<* *0.05) than the remaining arm muscles (Figs. 3 and 4). On the other hand, the peak increase in the Oxy‐Hb was not significantly different (*P *>* *0.05) among the distal arm muscles (triceps, 15 ± 4%; biceps, 17 ± 5%; ECR, 16 ± 4%, respectively). The Deoxy‐Hb of all arm muscles decreased (*P *<* *0.05) during the exercise (Fig. 3).

Passive one‐armed cranking significantly increased HR, SV, CO, and MAP (4 ± 1 beats/min, 4 ± 1 mL, 0.5 ± 0.1 L/min, and 6 ± 1 mmHg, respectively), but not TPR. Passive cranking had no or little influence on the Oxy‐Hb in the non‐contracting arm muscles but decreased (*P *<* *0.05) the Deoxy‐Hb (Fig. 3). The cardiovascular and NIRS responses and the mean RPE [6.5 (interquartile range, 6–7)] during passive cranking were much smaller (*P *<* *0.05) than those during voluntary cranking.

### Effects of autonomic blockades on the cardiovascular responses to voluntary arm exercise

**Figure 5:**
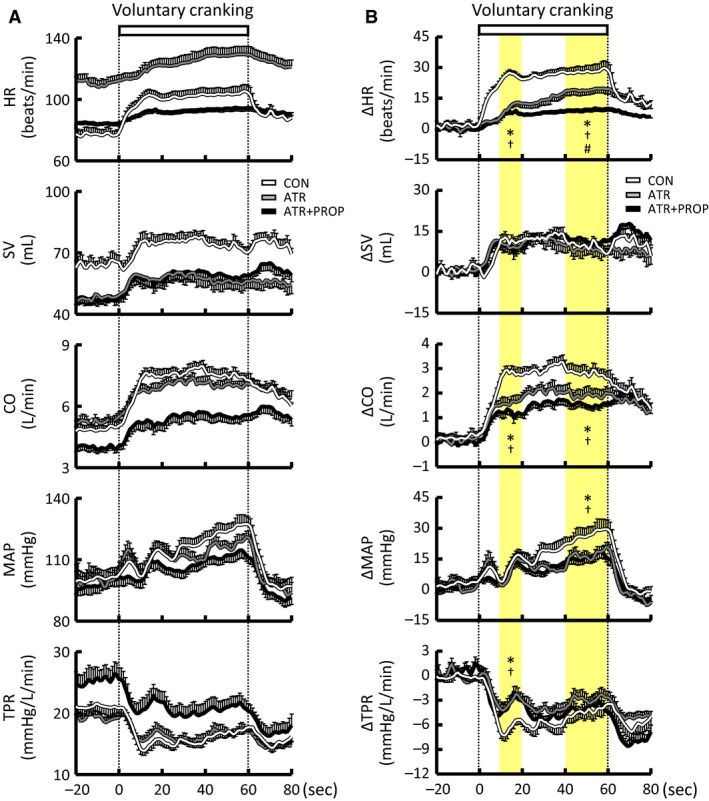
Effects of atropine on the time courses of the cardiovascular responses during voluntary one‐armed cranking in 10 subjects. (A) Absolute values. (B) Relative changes from the baseline levels. Atropine injection was followed by subsequent injection of propranolol. HR, heart rate. SV, stroke volume. CO, cardiac output. MAP, mean arterial blood pressure. TPR, total peripheral resistance. Each variable was sequentially calculated every 1 sec. Yellow areas indicate the early (10–20 sec) and later (40–60 sec) period of the exercise. White lines indicate the responses in the control condition without any drugs (CON). Gray lines indicate the responses in the atropine condition (ATR). Black lines indicate the responses in the atropine and propranolol condition (ATR+PROP). The relative changes during the early and later period of the exercise were compared among the conditions using a two‐way ANOVA with repeated measures and a Holm‐Sidak post hoc test. * Significant difference (*P *<* *0.05) between CON and ATR. † Significant difference (*P *<* *0.05) between CON and ATR+PROP. # Significant difference (*P *<* *0.05) between ATR and ATR+PROP. Values are mean ± SE.

**Figure 6:**
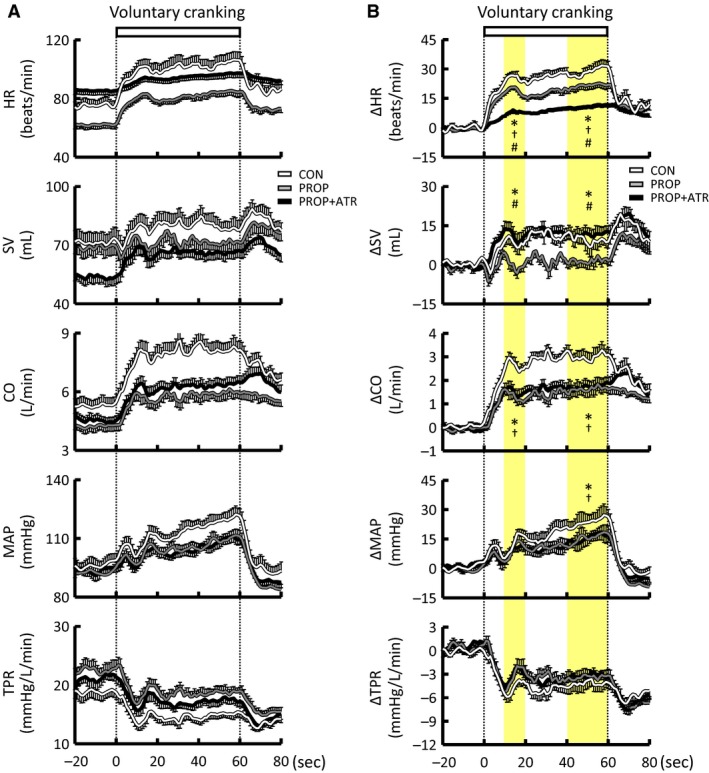
Effects of propranolol on the time courses of the cardiovascular responses during voluntary one‐armed cranking in 10 subjects. A: absolute values. B: relative changes from the baseline levels. Propranolol injection was followed by subsequent injection of atropine. Each variable was sequentially calculated every 1 sec. Yellow areas indicate the early (10–20 sec) and later (40–60 sec) period of the exercise. White lines indicate the responses in the control condition without any drugs (CON). Gray lines indicate the responses in the propranolol condition (PROP). Black lines indicate the responses in the propranolol and atropine condition (PROP+ATR). The relative changes during the early and later period of the exercise were compared among the conditions using a two‐way ANOVA with repeated measures and a Holm‐Sidak post hoc test. * Significant difference (*P *<* *0.05) between CON and PROP. † Significant difference (*P *<* *0.05) between CON and PROP+ATR. # Significant difference (*P *<* *0.05) between PROP and PROP+ATR. Values are mean ± SE.

The RPE was not changed by single administration of either atropine or propranolol but was increased by the combined blockades (Tables 1 and 2), although the exercise intensity was identical in all conditions. Atropine increased baseline HR and CO and decreased SV, while baseline MAP and TPR were not affected by atropine (Table 1 and Fig. 5). Propranolol decreased baseline HR and CO and increased TPR (*P *<* *0.05), while baseline SV and MAP were not affected by propranolol (Table 2 and Fig. 6). The combined blockades further influenced the baseline levels of the cardiovascular variables except for MAP, as compared to those with no drugs (Tables 1 and 2).

The initial and later increases in HR and CO and the initial decrease in TPR during voluntary one‐armed cranking were reduced by atropine (*P *<* *0.05), while the SV response was not altered by atropine (Fig. 5). Following propranolol, the initial and later increases in HR, SV, and CO during the exercise were reduced (*P *<* *0.05), while the decrease in TPR was not affected by propranolol (Fig. 6). Either atropine or propranolol did not change the initial increase in MAP but attenuated (*P *<* *0.05) the pressor response during the later period of exercise. The combined blockades further blunted the increase in HR during exercise (Figs. 5 and 6).

### Effect of atropine on the muscle oxygenation responses to voluntary arm exercise

**Figure 7:**
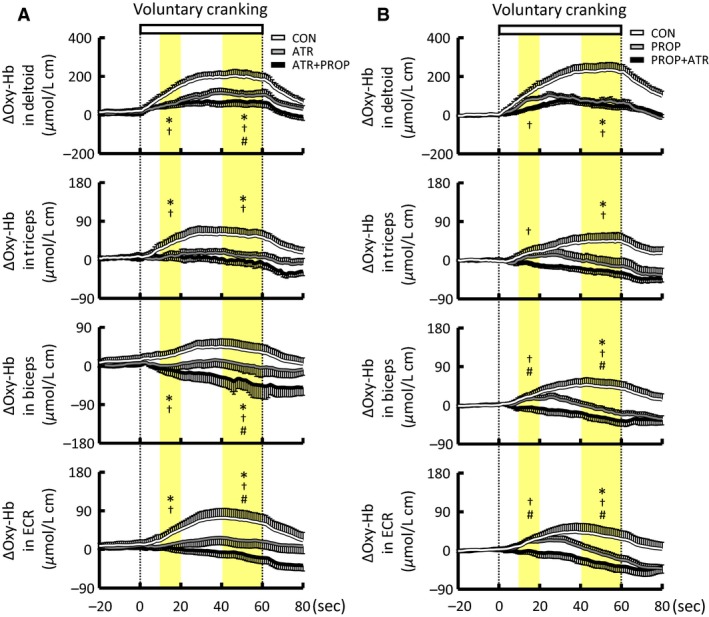
Effects of atropine and/or propranolol on the time courses of the Oxy‐Hb responses of the non‐contracting arm muscles during voluntary one‐armed cranking in 10 subjects. (A) Atropine injection was followed by subsequent injection of propranolol. (B) Propranolol injection was followed by subsequent injection of atropine. Each variable was sequentially calculated every 1 sec. Yellow areas indicate the early (10–20 sec) and later (40–60 sec) period of the exercise. White lines indicate the responses in the control condition without any drugs (CON). Gray lines indicate the responses in the atropine (A) or propranolol (B) condition (ATR or PROP). Black lines indicate the responses in the atropine and propranolol condition (ATR+PROP or PROP + ATR). The relative changes during the early and later period of the exercise were compared among the conditions using a two‐way ANOVA with repeated measures and a Holm‐Sidak post hoc test. * Significant difference (*P *<* *0.05) between CON and ATR or PROP. † Significant difference (*P *<* *0.05) between CON and ATR+PROP or PROP+ATR. # Significant difference (*P *<* *0.05) between ATR and ATR+PROP or between PROP and PROP+ATR. Values are mean ± SE.

**Figure 8:**
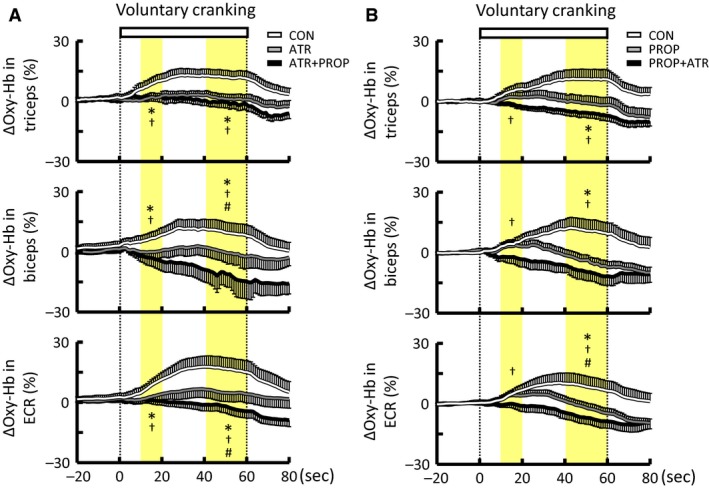
Effects of atropine and/or propranolol on the percent changes in Oxy‐Hb of the non‐contracting arm muscles during voluntary one‐armed cranking in 10 subjects. (A) Atropine injection was followed by subsequent injection of propranolol. (B) Propranolol injection was followed by subsequent injection of propranolol and atropine. The relative changes during the early and later period of the exercise were compared among the conditions using a two‐way ANOVA with repeated measures and a Holm‐Sidak post hoc test. * Significant difference (*P *<* *0.05) between CON and ATR or PROP. † Significant difference (*P *<* *0.05) between CON and ATR+PROP or PROP+ATR. # Significant difference (*P *<* *0.05) between ATR and ATR+PROP or between PROP and PROP+ATR. Values are mean ± SE.

Atropine did not change the baseline Oxy‐Hb of the arm muscles (Table 1). The initial increases in Oxy‐Hb of the arm muscles at the early period of voluntary one‐armed cranking were attenuated or abolished by atropine (*P *<* *0.05) (Fig. 7A). The attenuated Oxy‐Hb responses were sustained until the end of exercise. The subsequent combined blockades with atropine and propranolol decreased the baseline Oxy‐Hb of the triceps and biceps muscles (Table 1). The combined blockades did not further affect (*P *>* *0.05) the initial Oxy‐Hb responses of all arm muscles but decreased (*P *<* *0.05) the later Oxy‐Hb responses of the deltoid, biceps, and ECR muscles (Fig. 7A). The effects of atropine and the combined blockades on the Oxy‐Hb responses were confirmed by means of a percentage form (Fig. 8A). The reduction of Deoxy‐Hb in the arm muscles during the exercise was not affected by atropine, while the combined blockades blunted the later decrease of Deoxy‐Hb in the deltoid and triceps muscles (the data are not shown).

### Effect of propranolol on the muscle oxygenation responses to voluntary arm exercise

Propranolol decreased the baseline Oxy‐Hb of the triceps and ECR muscles (Table 2). The initial increases in Oxy‐Hb of the arm muscles at the early period of exercise were not affected by propranolol (*P *>* *0.05) (Fig. 7B). In contrast, the increases in Oxy‐Hb during the later period of exercise were attenuated or abolished by propranolol (*P *<* *0.05). Following subsequent combined blockades, the baseline Oxy‐Hb of the ECR muscle returned to the control level with no drugs (Table 2). The combined blockades abolished the initial increase of Oxy‐Hb in the biceps and ECR muscles (*P *<* *0.05) (Fig. 7B). The effects of propranolol and the combined blockades on the Oxy‐Hb responses were confirmed by means of a percentage form (Fig. 8B). The later decrease in the Deoxy‐Hb of the deltoid muscle was attenuated by propranolol, although propranolol did not affect the decreased Deoxy‐Hb of the remaining distal muscles (the data are not shown).

### The drug‐sensitive components of the Oxy‐Hb responses to voluntary one‐armed cranking

**Figure 9:**
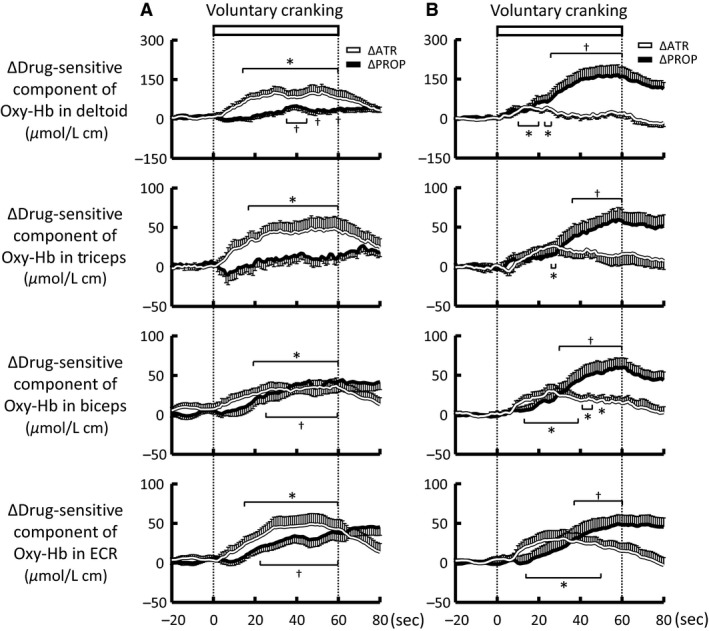
Time courses of the individual drug‐sensitive components of the Oxy‐Hb responses during voluntary one‐armed cranking in 10 subjects. (A) Atropine injection was followed by subsequent injection of propranolol. (B) Propranolol injection was followed by subsequent injection of atropine. An individual drug sensitive component was calculated by a difference in the Oxy‐Hb responses between the control (without any drug) and atropine or propranolol conditions, and by a difference in the Oxy‐Hb responses between the atropine or propranolol and the combined blockade conditions. White lines indicate the atropine‐sensitive component of the Oxy‐Hb response, while black lines indicate the propranolol‐sensitive component. Values are mean ± SE. * Significant difference (*P *<* *0.05) in the atropine‐sensitive component from the baseline. † Significant difference (*P *<* *0.05) in the propranolol‐sensitive component from the baseline.

Figure 9 summarizes the individual drug sensitive components of the Oxy‐Hb responses in the non‐contracting arm muscles during voluntary one‐armed cranking. The atropine‐sensitive component (as a difference in the Oxy‐Hb responses between the control and atropine conditions) was significantly (*P *<* *0.05) developed at 11–19 sec from the onset of exercise, while the propranolol‐sensitive component (as a difference in the Oxy‐Hb responses between the control and propranolol conditions) was developed (*P *<* *0.05) at 22–37 sec from the exercise onset. In the deltoid and triceps muscles, the atropine‐sensitive component (Fig. 9B), calculated by a difference in the Oxy‐Hb responses between the propranolol and combined blockade conditions, was smaller (*P *<* *0.05 in the deltoid, *P *=* *0.07 in the triceps) than the atropine‐sensitive component as a difference between the control and atropine conditions (Fig. 9A). Thus, the effects of atropine on the Oxy‐Hb responses were weakened following propranolol. Similarly, following pretreatment with atropine, the effects of propranolol were weakened (*P *<* *0.05) in the deltoid and triceps muscles (Fig. 9).

### The muscle oxygenation responses to mental imagery of voluntary one‐armed cranking

**Figure 10:**
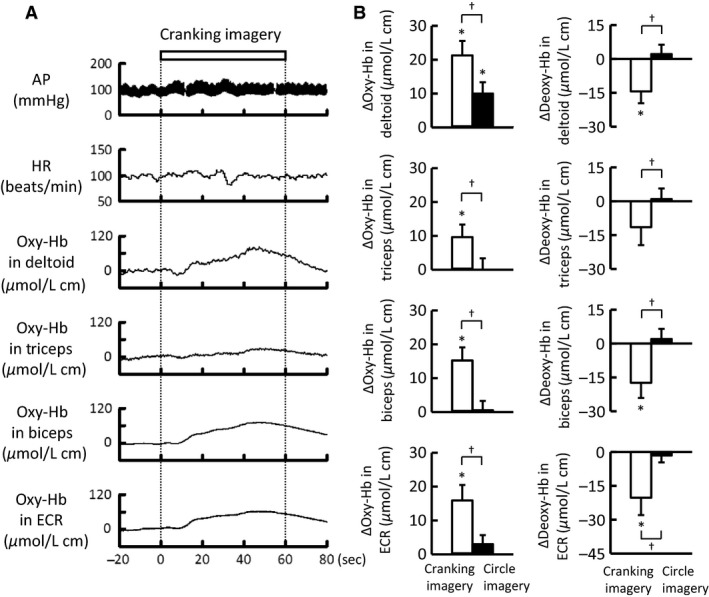
(A) Representative recordings of arterial blood pressure (AP), HR, and the Oxy‐Hb of the non‐contracting arm muscles during mental imagery of voluntary one‐armed cranking in a subject. (B) The average changes of the Oxy‐ and Deoxy‐Hb in the non‐contracting arm muscles during cranking‐ (□) and circle imagery (■) in 13 subjects. Values are mean ± SE. * Significant difference (*P *<* *0.05) from the baseline. † Significant difference (*P *<* *0.05) between the two imagery tasks.

Figure 10 shows the representative and averaged Oxy‐Hb responses of the non‐contracting arm muscles during cranking imagery. Cranking imagery increased (*P *<* *0.05) the Oxy‐Hb and decreased the Deoxy‐Hb of the arm muscles. The increase in Oxy‐Hb of the deltoid muscle tended to be greater than those of the remaining arm muscles (Figs. 4B and 10). In contrast, circle imagery failed to alter the Oxy‐ and Deoxy‐Hb of the arm muscles, although the Oxy‐Hb of the deltoid muscle increased slightly (Fig. 10). The vividness score was not different between cranking and circle imagery; the average score was 5.8 (interquartile range, 4–7) and 5.7 (interquartile range, 5–6), respectively. Slight increases (*P *<* *0.05) in HR, CO, and MAP (2 ± 1 beats/min, 0.2 ± 0.1 L/min, and 3 ± 1 mmHg, respectively) were observed during cranking imagery, but not circle imagery.

## Discussion

This study has examined whether central command increases oxygenation in the non‐contracting arm muscles via neurally mediated vasodilatation during contralateral upper limb exercise and to what extent the oxygenation response varies among the individual muscles of the upper limb. The major findings of this study are that (1) voluntary one‐armed cranking increased the Oxy‐Hb and decreased the Deoxy‐Hb of the non‐contracting arm muscles; (2) the increase in the Oxy‐Hb during one‐armed cranking was similar among the biceps, triceps, and ECR muscles, while that was the greatest in the deltoid muscle; (3) the oxygenation response of the arm muscles involved the rapid atropine‐sensitive and delayed propranolol‐sensitive components; (4) mental imagery of the one‐armed cranking increased the Oxy‐Hb of the arm muscles and decreased the Deoxy‐Hb; (5) passive one‐armed cranking did not increase the Oxy‐Hb of the non‐contracting arm muscles but decreased the Deoxy‐Hb. The new findings suggest that at the early period of voluntary one‐armed cranking, central command increases the oxygenation of the non‐contracting arm muscles via the sympathetic cholinergic vasodilator system. The centrally induced increase in muscle oxygenation may not be different among the distal arm muscles but may augment in the deltoid muscle.

### Increased muscle oxygenation by rapid cholinergic vasodilator mechanism

The increased oxygenation in the non‐contracting arm muscles during voluntary one‐armed cranking is expected to reflect an increase in tissue blood flow, because the EMG activity of the non‐contracting muscles was absent or negligible as shown in Figure 1 and thereby oxygen consumption was probably constant and at the minimum. The increase in oxygenation due to increased blood flow might occur in the muscle tissue rather than in the skin tissue, because cutaneous blood flow and vascular conductance of the forearm and shoulder were unchanged or decreased during static handgrip or dynamic leg exercise (Smolander et al. 1987; Taylor et al. 1988; Saad et al. 2001; Yanagimoto et al. 2003). The increase in muscle oxygenation is unlikely to result from a rise in perfusion pressure and/or redistribution of a rise in CO, because the increases in MAP and CO did not always increase the Oxy‐Hb of the arm muscles. For example, at the resting condition, fluctuation of MAP (~5–15 mmHg) did not change the Oxy‐Hb (data are not shown). MAP and CO always increased during voluntary one‐armed cranking but the changes in Oxy‐Hb varied among the muscles. Furthermore, administration of atropine did not affect both baseline MAP and the initial pressor response (Fig. 5) but blunted the initial increase in Oxy‐Hb during the exercise (Figs. 7 and 8). These findings suggest that the initial increase in oxygenation during the exercise is not attributed to the increases in MAP and CO but is caused by activation of sympathetic cholinergic vasodilator fibers (Matsukawa et al. 2013; Vianna et al. 2015). Although the present study could not prove vasodilatation in the arm muscles as a conductance value, our notion is supported by a previous study demonstrating that intraarterial injection of atropine blunted a decrease in resistance of the non‐exercising forearm during contralateral handgrip exercise (Sanders et al. 1989). Muscle sympathetic cholinergic nerve activity has not been recorded in humans, because sympathetic cholinergic fibers are normally silent (Horeyseck et al. 1976; Dean and Coote 1986) and cannot be detected using the conventional recording procedure for muscle sympathetic nerve activity (Delius et al. 1972).

On the other hand, propranolol had no effect on the initial increase in the Oxy‐Hb of the non‐contracting arm muscles at the early period of exercise (Figs. 7 and 8), suggesting no contribution of a *β*‐adrenergic mechanism to the initial increase in muscle oxygenation (Ishii et al. 2014). However, since the subsequent increase in Oxy‐Hb of the arm muscles was blunted or abolished by propranolol, it is suggested that *β*‐adrenergic vasodilatation in the arm muscles may develop as exercise proceeds. The similar observation in the non‐exercising forearm has been reported using intraarterial injection of propranolol (Eklund and Kaijser 1976). Epinephrine secreted from the adrenal medulla by activation of adrenal preganglionic sympathetic nerve is likely to evoke the delayed *β*‐adrenergic vasodilatation (Tsuchimochi et al. 2010), since the latent time of the propranolol‐sensitive oxygenation response (range, 22–37 sec) is coincident with the estimated transit time of epinephrine from the adrenal medulla to arterial blood vessels of the upper arm (range, 24–38 sec).

It is noted that the effect of one individual drug (atropine or propranolol) on the Oxy‐Hb response was weakened following pretreatment of the other drug (Figs. 7, 8, and 9). It is known that in the cardiac atrium, trachea, and ileum, activation of muscarinic receptors is modified by pre‐ and/or post‐junctional *β*‐adrenergic receptors and vice versa (Furukawa and Levy 1984; Reddy et al. 1995; Zhang et al. 1995). The mutual inhibition between the two vasodilator mechanisms may be the characteristics of the arm muscles rather than a leg muscle, because the two vasodilator mechanisms cooperated to produce increased oxygenation of the vastus lateralis muscle with a summative manner during one‐legged cycling (Ishii et al. 2014). Despite the inhibitory interaction, the subsequent combined blockades revealed the two distinct components of the Oxy‐Hb response, that is, the rapid atropine‐ sensitive and delayed propranolol‐sensitive components (Fig. 9).

### Differential oxygenation responses among non‐contracting skeletal muscles

Comparison of the Oxy‐Hb responses among the different muscular sites is considered to be permissible due to following reasons. First, thickness of skin and fat tissue was similar among the individual sites. Second, the skin blood flow response during exercise would be similar among the individual sites (Smolander et al. 1987). Third, the microvascular density per unit area may not differ greatly among the arm muscles, because type I muscle fibers, which have a denser capillary network than type II fiber, distribute at the same ratio in the arm muscles (approximately 53–61% in the deltoid; 32–62% in the triceps; 41–51% in the biceps; 62% in the ECR) (Johnson et al. 1973; Elder et al. 1982; Fugl‐Meyer et al. 1982). Fourth, when comparing the NIRS signals of biceps and ECR muscles between the two different interprobe distances (3 and 4 cm) in five subjects, the NIRS signals were not influenced by the difference in interprobe distance (unpublished data). Finally, different hydrostatic pressure to individual arm muscles is unlikely to affect the oxygenation responses, because we found the same Oxy‐Hb responses between the upper arm and forearm muscles (Figs. 7 and 8) and hydrostatic pressure influences arterial and venous blood vessels to the same extent.

Along this line, the comparison of the Oxy‐Hb response to voluntary one‐armed cranking revealed the greatest increase in oxygenation in the deltoid muscle (Fig. 4). Since the oxygenation response in the deltoid muscle involved the greater atropine‐ and propranolol‐sensitive components as compared to the remaining arm muscles (Fig. 9), the augmented sympathetic cholinergic and *β*‐adrenergic vasodilatation is likely to occur due to greater activation of the sympathetic cholinergic fibers and/or higher expression of the muscarinic and *β*‐adrenergic receptors in the muscle. Mishra and Haining (1980) reported using a hydrogen electrode technique that the increased blood flow immediately after upper limb exercise was greater in the deltoid than the biceps muscle. Thus, the greater cholinergic and *β*‐adrenergic vasodilatation might contribute to the augmented increase in oxygen supply to the contracting deltoid muscle as well.

### Centrally induced muscle oxygenation response by motor imagery

It is known that motor imagery activates the similar neural circuits with those activated by actual exercise, involving the dorsolateral prefrontal cortex, the insular cortex, and the anterior cingulate cortex (Decety 1996; Thornton et al. 2001; Williamson et al. 2002). Mental imagery of voluntary one‐armed cranking increased oxygenation in the arm muscles, whereas circle imagery did not (Fig. 10). This result suggests that activation of central “exercise‐related” circuits plays a role in increasing oxygenation in the arm muscles. However, it is not explicit whether central descending signal is identical between voluntary one‐armed exercise and its mental imagery, because the time courses and magnitudes of the Oxy‐Hb, as well as the cardiovascular responses, were different between the two tasks. Despite the above limitation, the mental imagery data seem important to identify central control of muscle oxygenation. It is of interest that the oxygenation response pattern caused by cranking imagery (i.e., a greater increase in oxygenation in the deltoid muscle than the remaining arm muscles) resembles the oxygenation response pattern caused by voluntary one‐armed cranking (Fig. 4).

### Effects of feedback from limb mechanoreceptors

Passive limb movement was utilized to isolate an influence of muscle mechanoreflex on the muscle oxygenation, although passive exercise cannot fully mimic the mechanical event and muscular tension during voluntary exercise. We found that activation of mechanosensitive afferents by passive one‐armed cranking had no or little influence on oxygenation in the contralateral non‐contracting arm muscles (Fig. 3). On the other hand, previous studies reported that passive one‐leg movement causes a slight vasodilatation and increase in oxygenation in the non‐exercising leg (McDaniel et al. 2010; Ishii et al. 2012). Taking the previous and present findings into account, it is suggested that muscle mechanoreflexes from upper and lower limb may have different influence on muscle oxygenation, although passive movement of either limb causes systemic cardiovascular changes to the same extent.

### Reduction in Deoxy‐Hb during voluntary one‐armed exercise and imagery

The decreased Deoxy‐Hb responses of the non‐contracting arm muscles may be determined by the changes in venous blood oxygenation and volume (Hoshi et al. 2001), because the degree of Deoxy‐Hb in arterial blood and the oxygen utilization of non‐contracting muscle are unlikely to change in this study. As another possible explanation for this, a rise in arterial blood supply may wash out venous blood and decrease venous blood oxygenation in the case that decreased Deoxy‐Hb is accompanied with increased Oxy‐Hb (as observed during voluntary cranking and imagery of the exercise). On the other hand, reduction of venous blood volume [e.g., venoconstriction (Bevegård and Shepherd 1966; Duprez et al. 1992; Hopman et al. 1993; Ooue et al. 2012)] may be reflexly evoked by mechanical motion of the upper limb, because passive cranking caused the reduction of Deoxy‐Hb with no changes of the Oxy‐Hb (Fig. 3).

### Limitations

Some fundamental limitations were involved in this study. First, skin blood flow was not measured in this study, although it is assumed that the blood flow responses in skin and fat tissues were similar in all sites where NIRS was measured and did not contribute to the increased Oxy‐Hb response. Second, the relative percent changes in the Oxy‐Hb of the deltoid muscle was not obtained, because blood supply to the area could not be stopped. Third, we did not adopt the use of conductance calculation from the ratio between Oxy‐Hb and MAP, because the Oxy‐Hb might not change in a simple linear fashion with perfusion pressure and the Oxy‐Hb responses were smaller than the responses in blood volume flow of the conduit artery (Fadel et al. 2004; Ishii et al. 2012, 2016). Fourth, when atropine or propranolol was injected into systemic circulation, each blocker blunted the increase in CO and the later pressor response during voluntary one‐armed cranking (Figs. 5 and 6). The blunted CO and pressor responses might account for the attenuated increases in Oxy‐Hb of the arm muscles. However, previous studies using plethysmography reported that the vasodilator response in the non‐exercising arm was actually blunted following intraarterial injection of atropine (Sanders et al. 1989) or propranolol (Eklund and Kaijser 1976). We found that the second combined injection of atropine and propranolol did not affect (*P *>* *0.05) the increases in CO and MAP but did blunt the increase in Oxy‐Hb of the arm muscles during voluntary exercise. Thus, it is unlikely that the Oxy‐Hb response during exercise was simply altered due to the changes in cardiac output and perfusion pressure.

## Conclusion

We demonstrated that central command increases oxygenation in the non‐contracting arm muscles at the early period of dynamic one‐armed exercise via the rapid cholinergic vasodilator mechanism. Taking our previous (Ishii et al. 2012, 2013, 2014, 2016) and present studies into consideration, it is speculated that central command may transmit the sympathetic cholinergic vasodilator signal bilaterally to skeletal muscles involved during exercise, that is, central command may cause vasodilatation in the bilateral arm muscles during one‐armed exercise and the same may be true for the leg during one‐legged exercise. Such centrally induced vasodilator effect may not be different among the distal arm muscles but may augment in the deltoid muscle.

## Conflict of Interest

None declared.
